# Supplementary material for: Neurovascular EGFL7 regulates adult neurogenesis in the subventricular zone and thereby affects olfactory perception
Source: Nat Commun. 2017 Jun 28;8:15922. doi: 10.1038/ncomms15922 (PMC5493759; doi:10.1038/ncomms15922)
Supplement: Supplementary Information [file ncomms15922-s1.pdf]

Type of file: PDF
Size of file: 0 KB
Title of file for HTML: Supplementary Information
Description: Supplementary Figures and Supplementary Tables.

Type of file: AVI
Size of file: 0 KB
Title of file for HTML: Supplementary Movie 1
Description: **Lineage progression of WT aNSCs/NPCs.** This movie shows the prototypical SVZ-derived aNSCs lineage progression of WT aNSCs/NPCs *in vitro* (Ortega et al, Nature protocols, 2011). aNSCs divided asymmetrically with one cell progressing towards neurogenesis through several rounds of amplifying divisions (TAPs). The other cells acquired a flat, astroglial-associated morphology and remained quiescent until the end of the live imaging experiment.

Type of file: AVI
Size of file: 0 KB
Title of file for HTML: Supplementary Movie 2
Description: **Lineage progression of EGFL7$^{-/-}$ aNSCs/NPCs.** This movie shows the SVZ-derived aNSCs lineage progression of EGFL7$^{-/-}$ aNSCs/NPCs in vitro. Upon division, cells acquired a flat and "quiescent" astroglial morphology. However, one of the cells re-entered the cell cycle and, eventually, gave rise to neurons. This effect was never observed in WT aNSCs/NPCs. Data suggest EGFL7 is a factor needed to keep NSCs quiescent.

Type of file: XLSX
Size of file: 0 KB
Title of file for HTML: Supplementary Data 1
Description: MaxQuant output table with SILAC ratios and protein identifications.

Type of file: TXT
Size of file: 0 KB
Title of file for HTML: Supplementary Data 2
Description: Results of 1D enrichment of SILAC ratios comparing EGFL7$^{-/-}$ and WT neurospheres at d2 and d5. Results are derived from data in Supplementary Data file 1 (XPro).

Type of file: PDF
Size of file: 0 KB
Title of file for HTML: Peer Review File
Description:

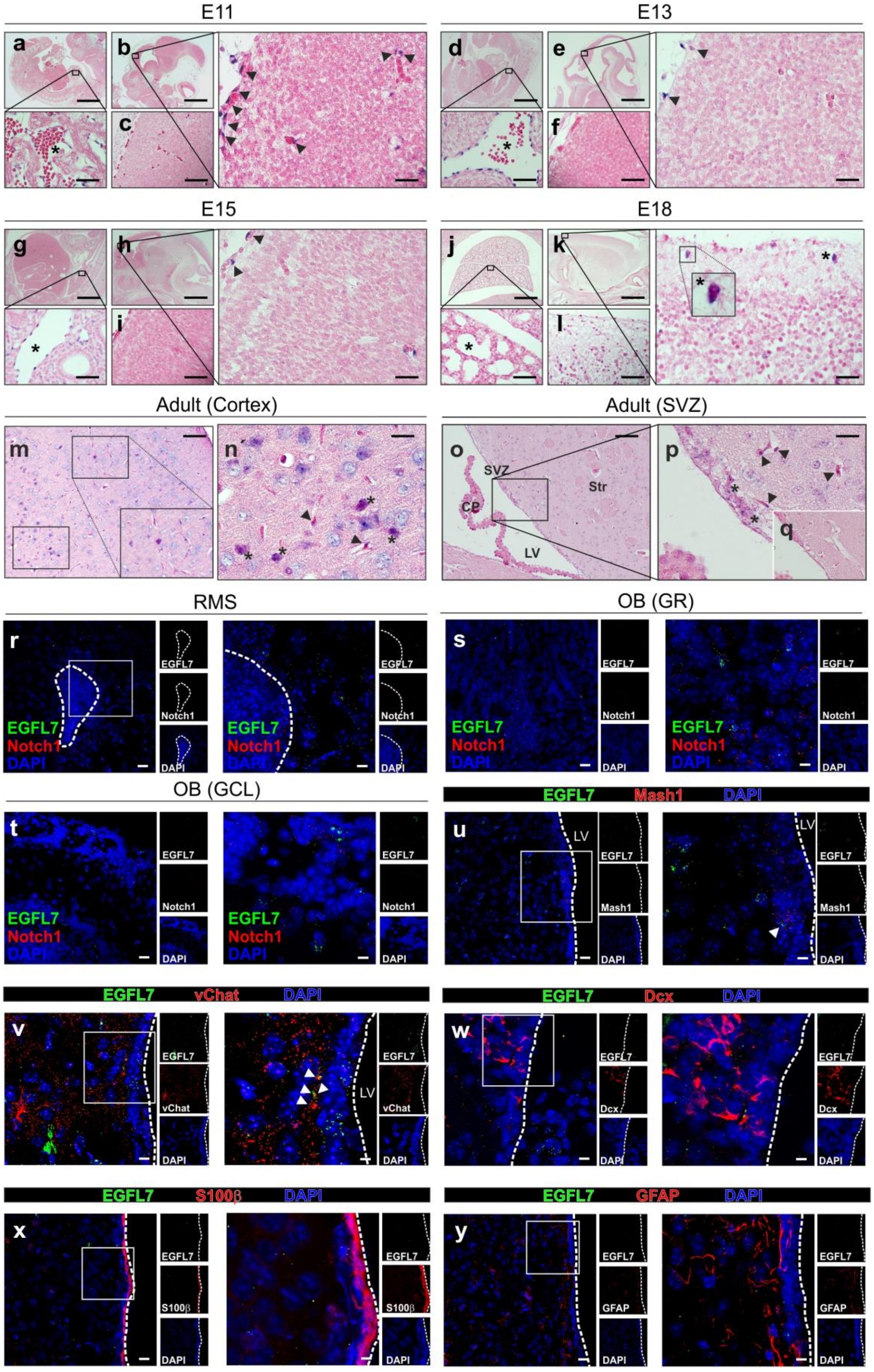
E11
E13
E15
E18
Adult (Cortex)
Adult (SVZ)
SVZ
CP
Str
LV
RMS
OB (GR)
OB (GCL)
EGFL7
Notch1
DAPI
Mash1
vChat
Dcx
S100β
GFAP

**Supplementary Figure 1. Localization of EGFL7 expression in the mouse brain and SVZ**

(**a-q**) *In situ* hybridization analyses revealed EGFL7 expression predominantly in *de novo* forming vessels of various organs, e.g., heart or lung (a, d, g, j, asterisks) or the developing brain (b, e, h, arrowheads). First EGFL7-positive non-ECs in the CNS were spotted at E18 within the marginal zone of the developing cortex (k, asterisks). In the adult brain, EGFL7 transcripts were detected in ECs (n, p, arrowheads) and non-ECs (n, p, asterisks) in the cortex and the SVZ. (c, f, i, l, q) Negative controls. (**r-y**) FISH analyses revealed EGFL7 transcripts in (s) the granular cell layer and (t) the glomerular cell layer of the OB, (r) but not in the RMS. The following markers were applied to discriminate among different cell types by FISH and IF analysis: Mash1 for TAPs, Dcx for NBs, ChAT/vChAT for projections of striatal neurons into the SVZ, GFAP for astrocytes and S100β for ependymal cells (Eps). EGFL7 expression in the SVZ was sporadically detectable in (u) $Mash1^+$ TAPs and (v) $ChAT/vChAT^+$ neuronal projections, whereas (w) $Dcx^+$ NBs, (x) $S100\beta^+$ Eps and (y) $GFAP^+$ astrocytes stained negative for EGFL7. Scale bars represent 1 mm (a, b, d, e, g, h, j, k), 100 µm (m, o), 50 µm (magnifications of a, d, g, j and c, f, I, l), 25 µm (magnifications of b, e, h, k and n, p, r-y) and 5 µm (magnifications of r-y).

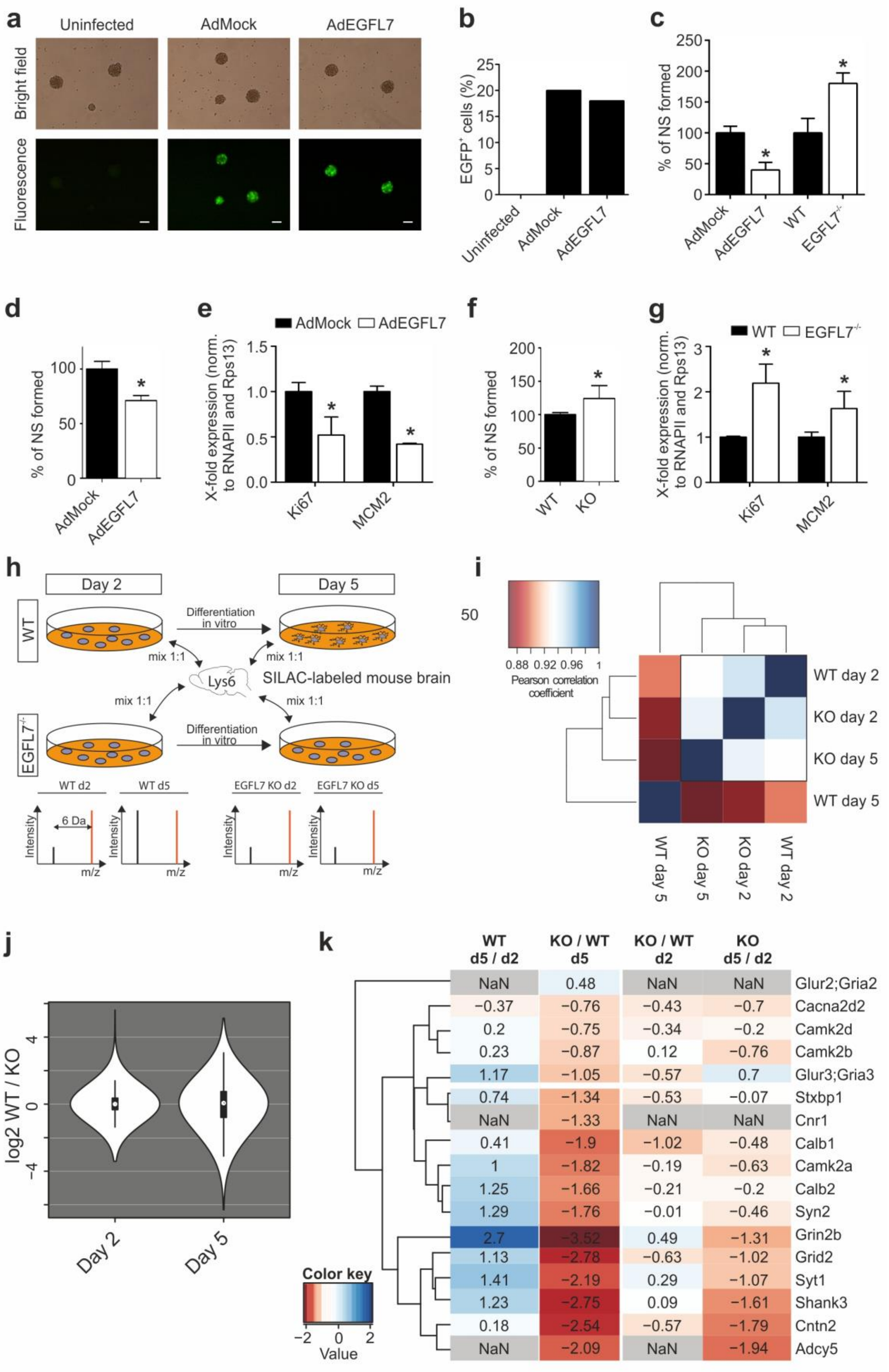
a
Uninfected
AdMock
AdEGFL7
Bright field
Fluorescence
b
EGFP+ cells (%)
Uninfected
AdMock
AdEGFL7
c
% of NS formed
AdMock
AdEGFL7
WT
EGFL7-/-
d
% of NS formed
AdMock
AdEGFL7
e
AdMock AdEGFL7
X-fold expression (norm. to RNAPII and Rps13)
Ki67
MCM2
f
% of NS formed
WT
KO
g
WT EGFL7-/-
X-fold expression (norm. to RNAPII and Rps13)
Ki67
MCM2
h
Day 2
Day 5
WT
Differentiation in vitro
mix 1:1
Lys6
SILAC-labeled mouse brain
EGFL7-/-
WT d2
WT d5
EGFL7 KO d2
EGFL7 KO d5
6 Da
Intensity
m/z
i
50
0.88 0.92 0.96 1
Pearson correlation coefficient
WT day 2
KO day 2
KO day 5
WT day 5
j
log2 WT / KO
Day 2
Day 5
k
WT d5 / d2
KO / WT d5
KO / WT d2
KO d5 / d2
NaN 0.48 NaN NaN Glur2;Gria2
−0.37 −0.76 −0.43 −0.7 Cacna2d2
0.2 −0.75 −0.34 −0.2 Camk2d
0.23 −0.87 0.12 −0.76 Camk2b
1.17 −1.05 −0.57 0.7 Glur3;Gria3
0.74 −1.34 −0.53 −0.07 Stxbp1
NaN −1.33 NaN NaN Cnr1
0.41 −1.9 −1.02 −0.48 Calb1
1 −1.82 −0.19 −0.63 Camk2a
1.25 −1.66 −0.21 −0.2 Calb2
1.29 −1.76 −0.01 −0.46 Syn2
2.7 −3.52 0.49 −1.31 Grin2b
1.13 −2.78 −0.63 −1.02 Grid2
1.41 −2.19 0.29 −1.07 Syt1
1.23 −2.75 0.09 −1.61 Shank3
0.18 −2.54 −0.57 −1.79 Cntn2
NaN −2.09 NaN −1.94 Adcy5
Color key
Value

**Supplementary Figure 2. EGFL7 affected SVZ-derived neurospheres**

(**a**) Adenovirus-infected neurospheres showing EGFP-expression. Scale bars represent 100 µm. (**b**) Comparable neurosphere infection rates were confirmed by FACS. (**c**) The self-renewal potential of neurospheres cultured under clonal conditions upon FACS sorting was reduced upon ectopic expression of EGFL7 (AdEGFL7) but increased upon *EGFL7*$^{-/-}$ and was therefore comparable to the results obtained by culturing neurospheres at clonal density. (**d**) Ectopic EGFL7 expression decreased the self-renewal potential (70.99 ± 8.1 versus (vs) 100 ± 12.04% in AdMock; n = 6; $P < 0.05$) and (**e**) the proliferation (qRT-PCR of Ki67; 0.52 ± 0.2 vs 1 ± 0.1-fold in AdMock; n = 3; $P < 0.05$ and MCM2; 0.42 ± 0.01 vs 1 ± 0.06-fold in AdMock; n = 3; $P < 0.05$) of neurospheres. (**f**) *EGFL7*$^{-/-}$ spheres displayed increased self-renewal potential (124.17 ± 43.38 vs 100 ± 6.9% in wild-type (WT) littermates; n = 6; $P < 0.05$) and (**g**) proliferation (qRT-PCR of Ki67; 2.19 ± 0.42 vs 1 ± 0.02-fold in WT; n = 3; $P < 0.05$ and MCM2; 1.63 ± 0.38 vs 1 ± 0.11-fold in WT; n = 3; $P < 0.05$). (**d-g**) verified data previously presented in Schmidt-MHH et al., Nat Cell Biol, 2009. (**h**) SILAC-based proteome analyses workflow using $^{13}C_6$ lysine = heavy Lys(6)-labeled mouse brain lysates as an internal standard. (**i**) Correlation matrix using Euclidean distance and Pearson correlation coefficients showing the similarity of the protein expression profiles of *EGFL7*$^{-/-}$ at d2, d5 and WT d2 but not WT d5. (**j**) Violin plot of log2 SILAC ratios comparing the protein profiles of *EGFL7*$^{-/-}$ and WT at d2 and d5. (**k**) Selected examples of neuronal proteins upregulated in d5 WT but not in d5 *EGFL7*$^{-/-}$ neurospheres. Statistics performed using Student's t-test/Mann Whitney *U*-test. Error bars indicate s.d.

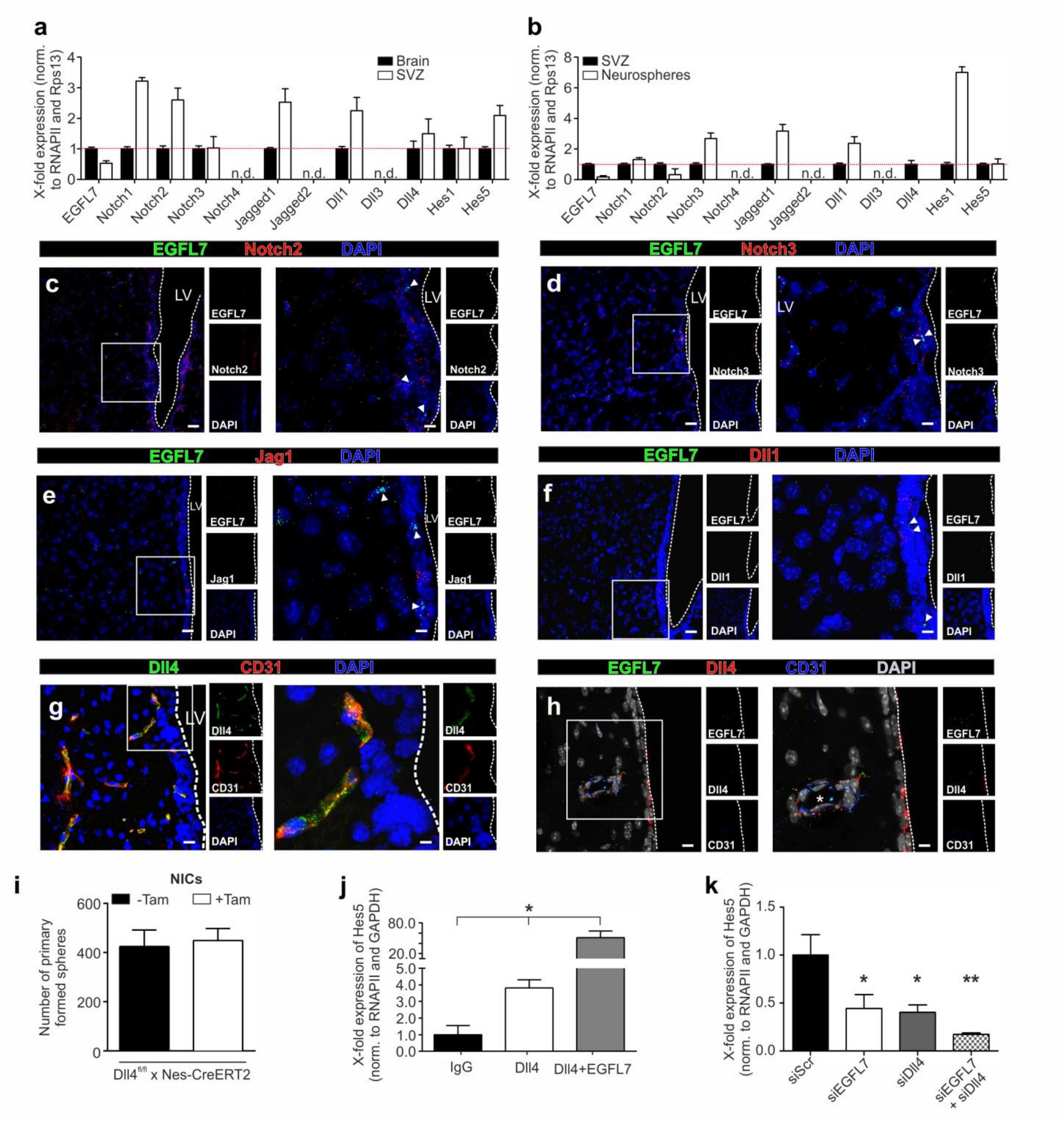
a
X-fold expression (norm. to RNAPII and Rps13)
Brain
SVZ
n.d.
EGFL7 Notch1 Notch2 Notch3 Notch4 Jagged1 Jagged2 Dll1 Dll3 Dll4 Hes1 Hes5
b
SVZ
Neurospheres
c EGFL7 Notch2 DAPI LV
d EGFL7 Notch3 DAPI LV
e EGFL7 Jag1 DAPI LV
f EGFL7 Dll1 DAPI
g Dll4 CD31 DAPI LV
h EGFL7 Dll4 CD31 DAPI
i NICs
-Tam
+Tam
Number of primary formed spheres
Dll4fl/fl x Nes-CreERT2
j
X-fold expression of Hes5 (norm. to RNAPII and GAPDH)
IgG
Dll4
Dll4+EGFL7
k
siScr
siEGFL7
siDll4
siEGFL7 + siDll4

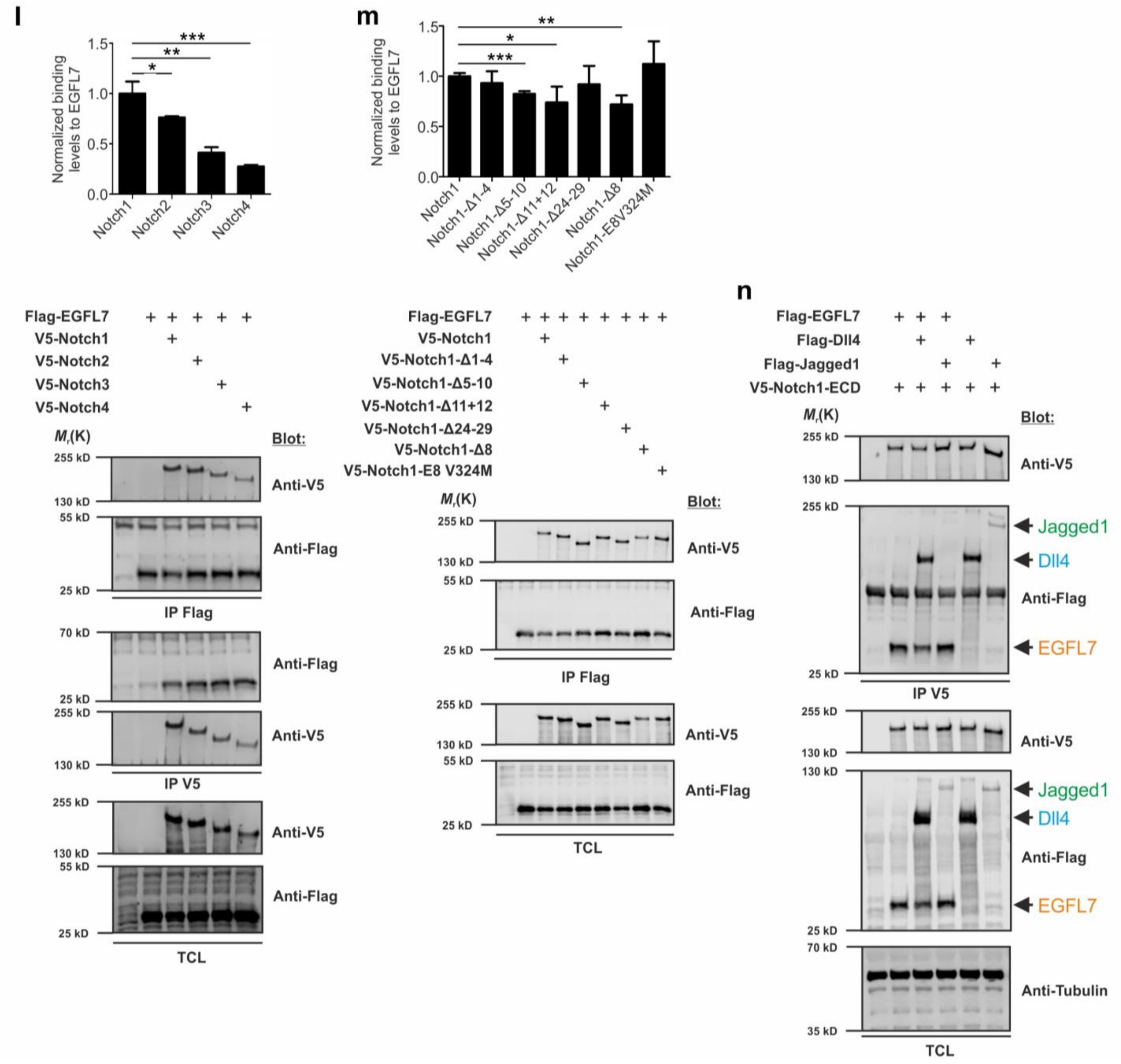


**Supplementary Figure 3. EGFL7 modified Notch signaling in the SVZ**

(**a**) qRT-PCR analyses of EGFL7 and various Notch pathway components in the SVZ *in vivo*. EGFL7 expression was reduced in the SVZ as compared to total brain extracts, whereas the levels of Notch1, Notch2, Jagged1, Dll4 and Hes5 expression were elevated. Notch4, Jagged2 and Dll3 expression were not detectable in both tissues. (**b**) qRT-PCR analysis of EGFL7 and various Notch

pathway components in neurospheres. EGFL7 and Notch2 expression were reduced in spheres as compared to SVZ tissue. Levels of Notch3, Jagged1, Dll1 and Hes1 expression were elevated in neurospheres. (**c-f**) Two-colored multiplex FISH of EGFL7 in combination with various Notch pathway components. EGFL7 transcripts were detected (c+d) in cells expressing Notch2/3 or in the close vicinity (arrowheads in magnifications), whereas (e) Jagged1 and (f) Dll1 transcripts were detectable exclusively in neighboring cells (arrowheads in magnifications). (**g**) IF of Dll4 and PECAM1 (CD31). Dll4 expression was found exclusively in $CD31^{+}$ blood vessels. (**h**) A combination of FISH and IF revealed co-localization of EGFL7 and Dll4 in blood vessels in the SVZ. LV, lateral ventricle. Scale bars represent 25 µm (c-h) and 5 µm (magnifications). (**i**) The stem cell-specific knock-out of Dll4 using $Dll4^{fl/fl}$*;Nestin-CreERT2* mice ($Dll4^{i\Delta NSC}$) did not affect the amount of primary neurospheres/NICs formed *in vitro* upon SVZ tissue microdissection. (**j+k**) qRT-PCR analyses of Hes5 as a marker for Notch signaling activity revealed (j) cooperative induction of Notch signaling by EGFL7 and Dll4 in neurospheres seeded on plastic dishes coated with the recombinant purified proteins (13.43x as compared to Dll4 alone; n = 3; $P < 0.0001$). (k) An additive reduction in Notch signaling in neurospheres was measured upon the knock-down of Dll4 or EGFL7 in co-cultured primary human umbilical vein endothelial cells (HUVECs) by respective siRNAs. (**l**) HEK293 cells were co-transfected with Flag-EGFL7 and V5-tagged extracellular domains (ECDs) of Notch1-4. Co-immunoprecipitation studies and quantitative western blots revealed differential binding of EGFL7 to Notch1-4. (**m**) Flag-EGFL7 was co-expressed along with V5-Notch1 ECD deletion mutants lacking EGF-like repeats important for Notch1 activation. Co-immunoprecipitation studies and quantitative western blots showed preferential binding of EGFL7 to EGF-like repeats in Notch1 involved in Notch receptor

activation *in trans* but not the ones related to inhibition *in cis*. (**n**) V5-Notch1-ECD and the Flag-tagged ligands EGFL7, Dll4 and Jagged1 were co-expressed in HEK293 cells. EGFL7 competed with Jagged1 for Notch1-ECD binding but cooperated with Dll4. Respective protein complexes were immunoprecipitated with anti-Flag or anti-V5 antibody in combination with agarose beads and were Western blotted with anti-Flag or anti-V5 antibodies. Detection and quantification were performed using an Odyssey quantification system (LI-COR). Statistics performed using Student's t-test/Mann Whitney *U*-test. Error bars indicate s.d.

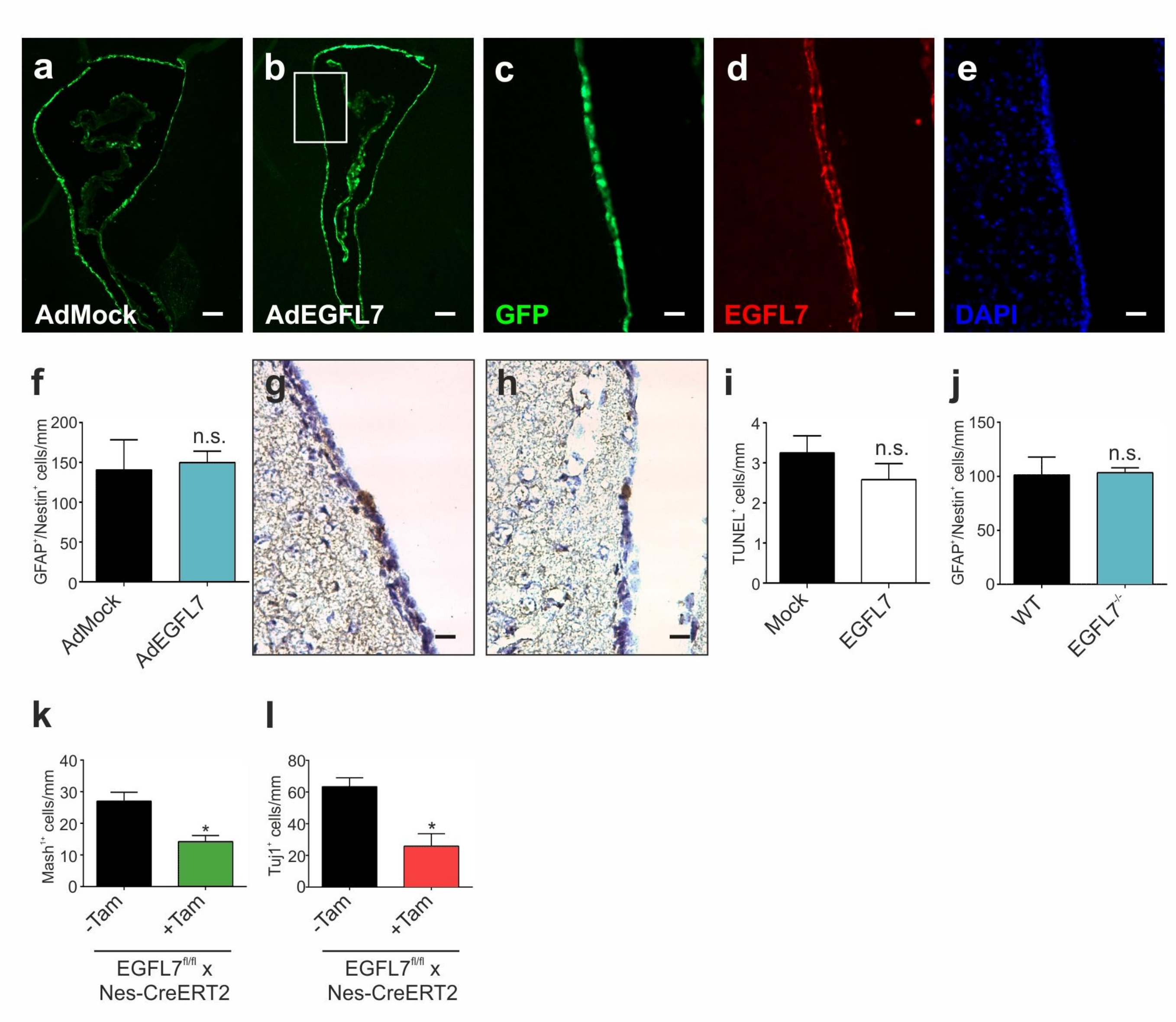


**Supplementary Figure 4. EGFL7 modulated the cytoarchitecture of the SVZ**

(**a-e**) Subsequent to CVI, successful infection of ependyma and SVZ was confirmed by IF of adenovirus encoded (a-c) EGFP and (d) ectopic EGFL7. (e) Cell nucleus staining. (**f**) The total number of GFAP$^+$/Nestin$^+$ aNSCs/NPCs was not altered by AdEGFL7 upon CVI (156.2 ± 15.74 vs 140.1 ± 11.98 cells/mm in AdMock; n = 6). (**g-i**) Apoptosis was not affected by ectopic EGFL7 expression as determined by TUNEL staining (g+h). (**j**) The total number of GFAP$^+$/Nestin$^+$

aNSCs/NPCs was not altered in *EGFL7*$^{-/-}$ mice (103.3 ± 4.73 vs 101.2 ± 16.53 cells/mm in WT; n = 6). The tamoxifen-induced EGFL7 knock-out in stem cells of adult *EGFL7*$^{fl/fl}$*;Nestin-CreERT2* mice (*EGFL7*$^{i\Delta NSC}$) at 12 weeks of age caused a decrease in (**k**) TAPs (14.23 ± 1.14 vs 27.1 ± 2.12 cells/mm in WT; n = 3; $P < 0.01$) and (**l**) NBs (; 25.85 ± 7.82 vs 63.37 ± 5.6 cells/mm in WT; n = 3; $P < 0.05$), thereby phenocopying the results obtained in constitutive *EGFL7*$^{-/-}$ mice. Statistics performed using Student's t-test/Mann Whitney *U*-test. Error bars indicate s.d. Scale bars represent 100 µm (a, b) and 25 µm (c, d, e, g, h).

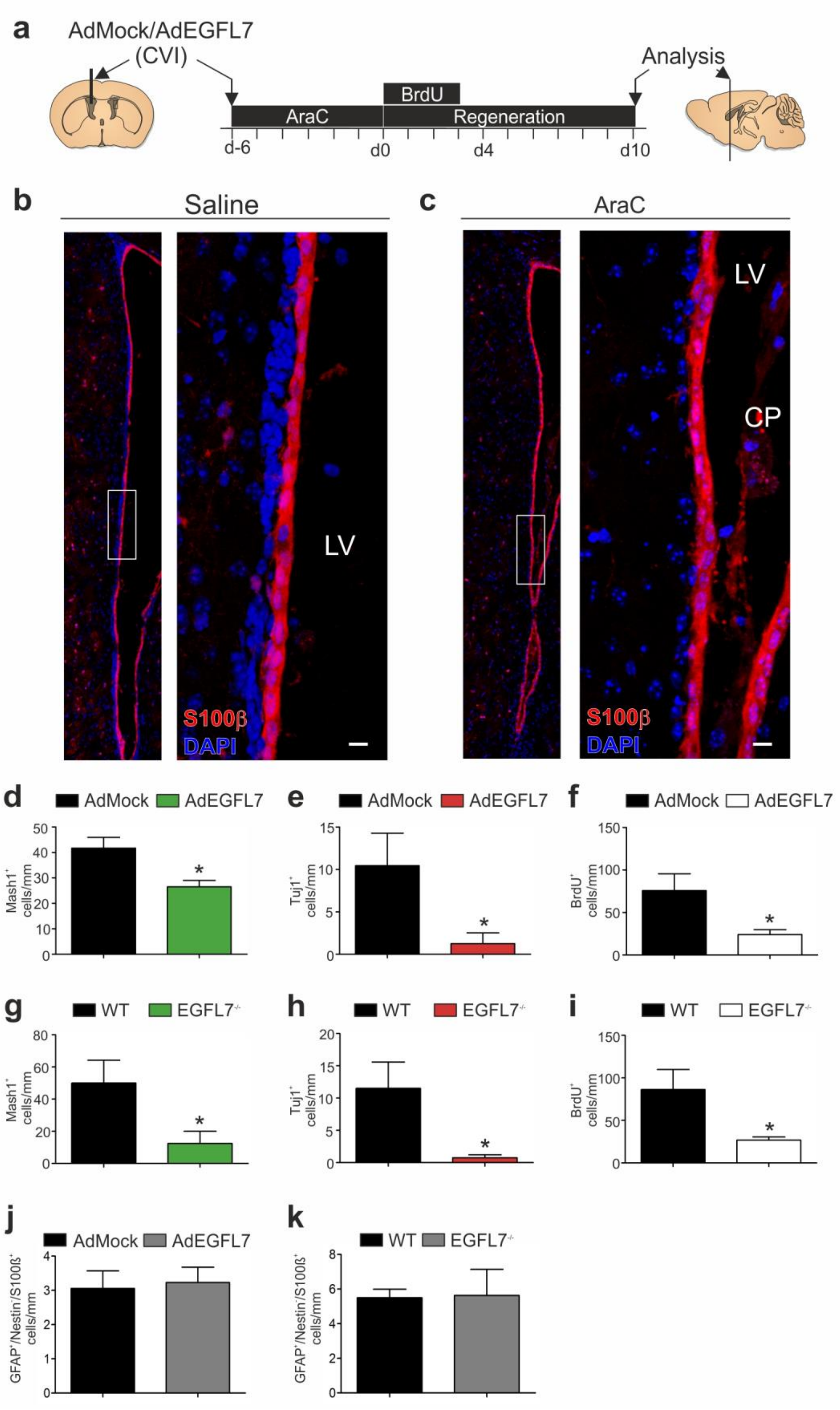

a
AdMock/AdEGFL7
(CVI)
Analysis
BrdU
AraC
Regeneration
d-6
d0
d4
d10
b
Saline
c
AraC
LV
CP
S100β
DAPI
d
AdMock
AdEGFL7
Mash1+ cells/mm
e
Tuj1+ cells/mm
f
BrdU+ cells/mm
g
WT
EGFL7-/-
h
i
j
GFAP+/Nestin+/S100ß+ cells/mm
k

**Supplementary Figure 5. EGFL7 changed the activation state of NSCs**

(**a**) Schematic of experimental set-up. (**b+c**) Depletion of SVZ was confirmed by IHC at day 0. (**d-i**) Quantification of NSC progeny in the SVZ 10 d after AraC-mediated deprivation of proliferating cells. (d-f) AdEGFL7 as well as (**g-i**) *EGFL7*$^{-/-}$ caused a delay in TAPs (AdEGFL7; 52.98 ± 5.04 vs 83.34 ± 8.44 cells/mm in AdMock; n = 6; $P < 0.01$, *EGFL7*$^{-/-}$;11.56 ± 6.75 vs 50.77 ± 14.28 cells/mm in WT; n = 6; $P < 0.05$), NBs (AdEGFL7; 1.26 ± 1.26 vs 10.45 ± 3.83 in AdMock; n = 6; $P < 0.05$, *EGFL7*$^{-/-}$; 0.75 ± 0.48 vs 11.5 ± 4.09 cells/mm in WT; n = 6; $P < 0.05$) and proliferating cells (AdEGFL7; 21.08 ± 2.46 vs 59.39 ± 15.7 cells/mm in AdMock; n = 6; $P < 0.05$, *EGFL7*$^{-/-}$; 26.95 ± 3.7 vs 86.27 ± 23.58 cells/mm in WT; n = 6; $P < 0.05$) repopulating the SVZ. (**j**) The amount of astrocytes (GFAP$^{+}$/Nestin$^{-}$/S100β$^{+}$) in the SVZ was not changed upon CVI of AdEGFL7 or (**k**) in *EGFL7*$^{-/-}$ mice. Statistics performed using Student's t-test/Mann Whitney *U*-test. Error bars indicate s.d. Scale bars represent 10 µm (b, c).

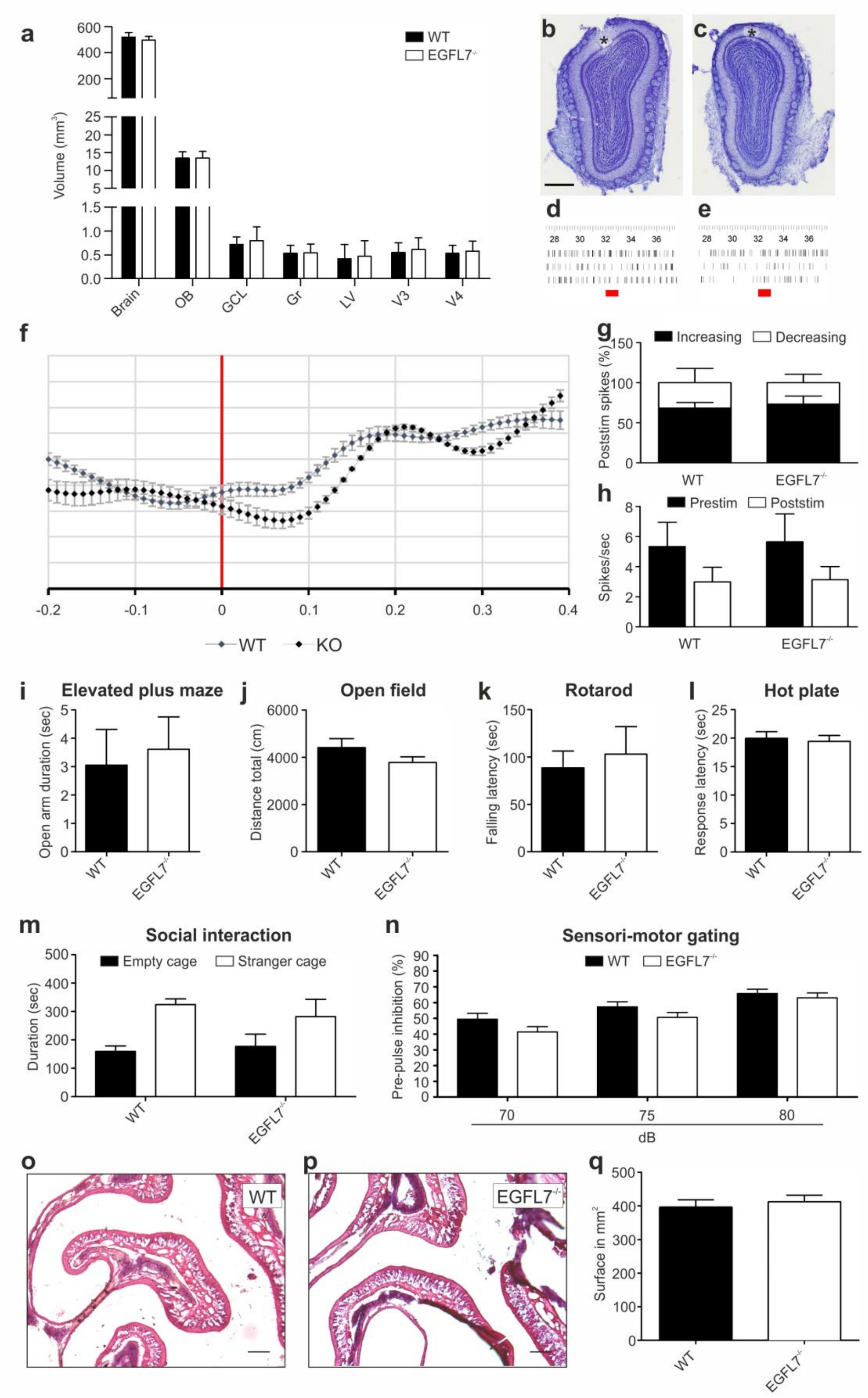
a
WT
EGFL7-/-
Volume (mm3)
600
400
200
25
20
15
10
5
1.5
1.0
0.5
0.0
Brain
OB
GCL
Gr
LV
V3
V4
b
c
d
e
28 30 32 34 36
f
-0.2 -0.1 0 0.1 0.2 0.3 0.4
WT
KO
g
Increasing
Decreasing
Poststim spikes (%)
150
100
50
0
WT
EGFL7-/-
h
Prestim
Poststim
Spikes/sec
8
6
4
2
0
WT
EGFL7-/-
i
Elevated plus maze
Open arm duration (sec)
WT
EGFL7-/-
j
Open field
Distance total (cm)
6000
4000
2000
0
k
Rotarod
Falling latency (sec)
150
100
50
0
l
Hot plate
Response latency (sec)
25
20
15
10
5
0
m
Social interaction
Empty cage
Stranger cage
Duration (sec)
500
400
300
200
100
0
n
Sensori-motor gating
Pre-pulse inhibition (%)
90
80
70
60
50
40
30
20
10
0
70
75
80
dB
o
WT
p
EGFL7-/-
q
Surface in mm2
500
400
300
200
100
0

**Supplementary Figure 6. EGFL7 altered neurogenesis and behavior**

(**a**) Magnet resonance imaging revealed no significant difference in the volume of *EGFL7*$^{-/-}$ brains or various subregions as compared to WT. OB, olfactory bulb; GCL, glomerular cell layer; Gr, granular cell layer; LV, lateral ventricle; V3, third ventricle; V4, fourth ventricle. (**b+c**) Nissl staining showing electrode penetration sites (asterisks) for electrophysiological experiments. Scale bar represents 1 mm. (**d+e**) Examples of raster plots of five neurons, one derived from a WT and the other from an *EGFL7*$^{-/-}$ mouse. Red bar indicates an odor stimulus of 1 sec. (**f**) A peristimulus time histogram (bin = 5 ms) showed that WT neurons rapidly increased their firing rate upon amyl acetate stimulus (red vertical line). *EGFL7*$^{-/-}$ neurons responded slower to the stimulus within 200 ms but thereafter increased their firing rate comparably to WT controls. (**g**) Histogram representing the distribution of neurons increasing or decreasing their firing rate in response to the stimulus in WT and *EGFL7*$^{-/-}$ mice. (**h**) Chart representing the extent of decrease in firing rate in WT (n = 27) and *EGFL7*$^{-/-}$ mice (n = 17). (**i-n**) Evaluation of basic behavioral functions in *EGFL7*$^{-/-}$ mice revealed no gross behavioral abnormalities. (i) Elevated plus maze test for anxiety. Y-axis: time spent in the open arms. (j) Open field test for general activity. Y-axis: total distance traveled. (k) Rotarod test for motor balance and coordination. Y-axis: latency to fall from the rod. (l) Hot plate test of pain threshold. Y-axis: latency to show a pain response. (m) Social novelty preference test for social behavior. Y-axis: time spent in different compartments. (n) Pre-pulse inhibition of startle response test for sensorimotor gating. Y-axis: percent of pre-pulse inhibition. Open bars - WT; filled bars - *EGFL7*$^{-/-}$ animals. No statistically significant differences between groups were revealed; n(WT) = 19, n(*EGFL7*$^{-/-}$) = 12. (**o+p**) HE-staining of the nasal cavities of *EGFL7*$^{-/-}$ and WT mice. (**q**) Quantification of the surface area of the main olfactory epithelium within the olfactory turbinates of EGFL7$^{-/-}$ and WT mice shows no difference; n(WT) = 3, n(*EGFL7*$^{-/-}$) = 3. Scale bars represent 100 µm.

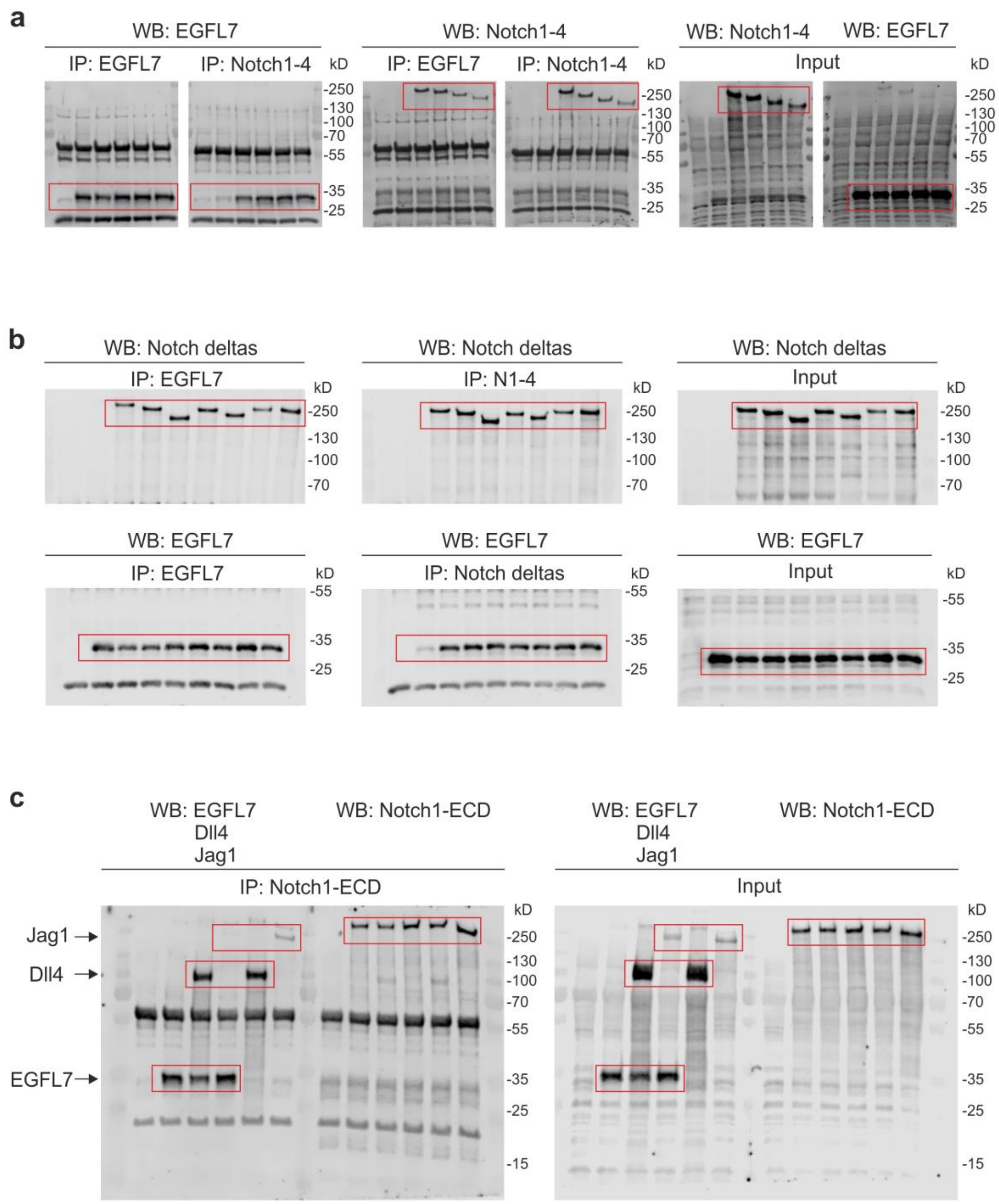


**Supplementary Figure 7. Uncropped western blot images of supplementary Fig. 3.**

**Supplementary Table 1.** Primers used for qRT-PCR

| Gene | ID number | Primer | Sequence (5´-3´) |
|---|---|---|---|
| mDll1 | NM_007865.3 | Dll1_for | GTCTGCCAGGGTGTGATGAC |
| | | Dll1_rev | GCAGGTGCCATGGAGACAAC |
| mDll3 | NM_007866.2 | mDll3_for | ACGCCATTCCCAGACGAGTG |
| | | mDll3_rev | TCCAGGCAACGGCATTCATC |
| mDll4 | NM_019454.3 | mDll4_for | GTTCTCGCCTATGCAAGAAG |
| | | mDll4_rev | CTCATGACAGCCAGAAAGAC |
| mEGFL7 | NM_178444.4 | EGFL7_for | CACCTACCGAACCATCTACC |
| | | EGFL7_rev | ACATGGAGGCTGGCATATTG |
| mGAPDH | NM_001289726.1 | GAPDH_for | TGAAGCAGGCATCTGAGGG |
| | | GAPDH_rev | CGAAGGTGGAAGAGTGGGAG |
| mHes1 | NM_008235.2 | mHes1_for | GAGGCGAAGGGCAAGAATAAA |
| | | mHes1_rev | GTGGACAGGAAGCGGGTC |
| mHes3 | NM_008237.4 | mHes3_for | AGCCTCTGATGGAGAAGAAG |
| | | mHes3_rev | CACTCAGCTCCAGGATATCG |
| mHes5 | NM_010419.4 | mHes5_for | TGGAGAAGGCCGACATCCTG |
| | | mHes5_rev | AGGAACTGTACCGCCTCCTG |
| mHey1 | NM_010423.2 | mHey1_for | CCGAAGTTGTCCGTTATCTG |
| | | mHey1_rev | GCTGGGATGCGTAGTTGTTG |
| mHey2 | NM_013904.1 | mHey2_for | CCAATTCACCGACAACTACC |
| | | mHey2_rev | AGCTGTTGGCACTAGTCTTC |
| mJagged1 | NM_013822.5 | mJagged1_for | GTCTCTGTCCCACTGGTTTC |
| | | mJagged1_rev | AGTCACTGGCACGATTGTAG |
| mJagged2 | NM_010588.2 | mJagged2_for | ATGCTGAGCCTGACCAATAC |
| | | mJagged2_rev | ACGGACAGTGGCATTCAAAG |
| mKi67 | NM_001081117.2 | mKi67_for | CAGACGAGCAAGAGACAAAG |
| | | mKi67_rev | TCAATCTGCGCTCTACCTAC |
| mMCM2 | NM_008564.2 | MCM2_for | CCGTTCCAAGGATGCCATTC |
| | | MCM2_rev | AAGCCGTTGGCGGTGTTAAG |
| mNotch1 | NM_008714.3 | mNotch1_for | CCTCAGATGGTGCTCTGATG |
| | | mNotch1_rev | CTCAGGTCAGGGAGAACTAC |
| mNotch2 | NM_010928.2 | mNotch2_for | TGGCTTCATCTGTCGTTGTC |
| | | mNotch2_rev | TCCCGAGTCAGTGTGGATAC |
| mNotch3 | NM_008716.2 | mNotch3_for | GACAGAGTCAATGGCTTCAG |
| | | mNotch3_rev | CTGCACAGCGACACTCATAG |
| mNotch4 | NM_010929.2 | mNotch4for | CACAGCCATCTGGCTACAAC |
| | | mNotch4rev | GCAGGAATAGCCCTCAGGAC |

| | | | |
|---|---|---|---|
| **mRNAPolII** | **NM_001291068.1** | **RNAPolII_for** | **GACAAAACTGGCTCCTCTGC** |
| | | **RNAPolII_rev** | **GCTTGCCCTCTACATTCTGC** |
| **mRps13_3** | **NM_026533.3** | **mRPS13_for** | **TTCACCGATTGGCTCGATAC** |
| | | **mRPS13_rev** | **TTATGCCACTAGAGCAGAGG** |
| **hDll4** | **NM_019074.3** | **hDll4_for** | **GCG AGA AGA AAG TGG ACA GG** |
| | | **hDll4_rev** | **ACA GTA GGT GCC CGT GAA TC** |
| **hEGFL7** | **NM_016215.4** | **hEGFL7_for** | **TGG ATG AAT GCA GTG CTA GG** |
| | | **hEGFL7_rev** | **CCT TGG GCA CAC AGA GTG TA** |
| **hHPRT1** | **NM_000194.2** | **hHPRT1_for** | **TGA GGA TTT GGA AAG GGT GT** |
| | | **hHPRT1_rev** | **GAG CAC ACA GAG GGC TAC AA** |
| **hACTB** | **NM_001101.3** | **hACTB_for** | **CAT CAC CAT TGG CAA TGA GC** |
| | | **hACTB_rev** | **CGA TCC ACA CGG AGT ACT TG** |

**Supplementary Table 2.** Antibodies used for IF, ISH, WB or IP

| Antibody/ Antigen | Applications | Dilution | Source | Company | Order ID |
|---|---|---|---|---|---|
| Actin | W | 1:1000 | m (mc) | Santa Cruz | sc-8432 |
| Digoxyn (Alkalyne Phosphatase conj.), DIG-AP | ISH | 1:2000 | sh (pc) | Roche | 11093274910 |
| EGFL7 (58) | IHC | 1:50 | rb (pc) | Reliatec | not comm. available |
| EGFL7 (R-12) | W<br>IP<br>IHC | 1:75<br>1:1000<br>1:50 | gt (pc) | Santa Cruz | sc-34416 |
| Glial Fibrillary Acidic Protein, GFAP (G-A-5) | IHC | 1:400 | m (mc) | Millipore | MAB3402 |
| Green Fourescent Protein (Alexa488 conj.), GFP-488 | IHC | 1:100 | rb (pc) | Molecular Probes | |
| Ki-67 (TEC-3) | IHC<br>ICC (FACS) | 1:50 | rt (mc) | Dako | M7249 |
| NeuN | IHC | 1:100 | m (mc) | Millipore | MAB377 |
| Neuronal Class III β-Tubulin (TUJ1) | IHC | 1:500 | m (mc) | Covance | MMS-435P |
| PECAM-1/CD31 (MEC13.3) | IHC | 1:200 | rt (mc) | BD Biosciences | 557355 |
| Tubulin | W | 1:7000 | m (mc) | Lab Vision | MS-581-PO |
| V5-Tag | W | 1:5000 | m (mc) | Invitrogen | |
| V5-Tag | IP | 1:250 | m (mc) | Biozol | BZL04627 |

**Applications Key:** W-Western, IP-Immunoprecipitation, IHC-Immunohistochemistry, ICC-Immunocytochemistry, ISH-*In situ* Hybridization
**Species Key:** gt-goat, m-mouse, rb-rabbit, rt-rat, sh-sheep (mc-monoclonal, pc-polyclonal)
